# Orientation Uncertainty Characteristics of Some Pose Measuring Systems

**DOI:** 10.1155/2017/2696108

**Published:** 2017-12-31

**Authors:** Marek Franaszek, Geraldine S. Cheok

**Affiliations:** National Institute of Standards and Technology, Gaithersburg, MD 20899, USA

## Abstract

We investigate the performance of pose measuring systems which determine an object’s pose from measurement of a few fiducial markers attached to the object. Such systems use point-based, rigid body registration to get the orientation matrix. Uncertainty in the fiducials’ measurement propagates to the uncertainty of the orientation matrix. This orientation uncertainty then propagates to points on the object’s surface. This propagation is anisotropic, and the direction along which the uncertainty is the smallest is determined by the eigenvector associated with the largest eigenvalue of the orientation data’s covariance matrix. This eigenvector in the coordinate frame defined by the fiducials remains almost fixed for any rotation of the object. However, the remaining two eigenvectors vary widely and the direction along which the propagated uncertainty is the largest cannot be determined from the object’s pose. Conditions that result in such a behavior and practical consequences of it are presented.

## 1. Introduction

The pose of a rigid object is defined by six degrees of freedom (6DOF): three angles describing the object’s orientation matrix **R** and three components describing the object’s position ***τ*** (e.g., center of mass or center of bounding box). Accounting for the uncertainty of the measured pose is of great importance in many applications (e.g., propagating uncertainty along different joints in a robot arm or fusing measurements from multiple sensors) and it has been studied for a long time [1]. The methodology used in these studies is based on a 4 × 4 homogenous transformation matrix, related exponential mapping, and Lie algebra [2].

In this paper, our focus is on a different aspect of pose uncertainty. We are interested in how uncertainty of a single static measurement of a rigid body pose propagates to any Point of Interest (POI) associated with the object (e.g., a point on its surface). When the Computer Aided Design (CAD) model of an object is known, the location of any POI can be calculated using 6DOF data acquired by pose measuring systems [3]. In assembly applications where rigid parts need to be mated using autonomous robotic systems [4–8], uncertainty in pose has to be propagated to the POI. For example, in a peg-in-hole experiment (commonly used to test a robot’s performance [9–12]), uncertainty in the hole location directly affects the test outcome [13–17]. Thus, we acquire repeated measurements of a rigid object’s pose, obtained in the same experimental conditions, to investigate the uncertainty of a given POI.

In most practical applications, the six components of pose are not directly measured but are derived from other raw measurements. Many pose measuring systems report 6DOF data of an object based on the measurement of the 3D positions of a few points. These points, also known as fiducial markers, are rigidly attached to (or around) the measured object. Some systems may not require the use of markers as they may be trained to use some characteristic features of the measured object (e.g., well defined corner points). For systems which use 3D points, a homogenous transformation {**R**, ***τ***} is found using point-based rigid body registration and minimizing the following error function called the Fiducials Registration Error (FRE)

(1) $$\text{FRE}\;(R,\tau )=\sqrt{\frac{1}{J}\sum_{n=1}^{J}{\left\|{\mathrm{RX}}_{n}+\tau -Y_{n}\right\|}^{2}},$$

where {**X**}*_J_* is a set of *J* fiducials measured in one coordinate frame (working frame) and {**Y**}*_J_* is a set of corresponding fiducials measured in the second frame (destination frame). Pose measuring systems track the movement of the working frame and the transformation {**R**, ***τ***} defines the object’s 6DOF pose relative to a starting reference frame (coordinate frame associated with the instrument). Point-based rigid body registration not only is implemented in pose measuring systems but is commonly used in many field applications where 3D points are measured in one frame but have to be accessed in another frame where they are required. These points (targets) need to be transformed from the working frame to the destination frame using the previously determined transformation {**R**, ***τ***}.

Noise present in the measurement of the fiducials propagates to the transformation {**R**, ***τ***}. Such noisy transformation (when applied to a target) yields random deviation of the transformed target from its nominal true location **r** and is quantified by the Target Registration Error (TRE(**r**)) defined as the root mean square of distances between these two points. The development of a closed form equation for TRE has been the aim of extensive research for many years [18–22]. The main conclusions from these efforts can be summarized as follows: (1) TRE(**r**) depends on the location of target **r** relative to the three main axes of the moment of inertia derived from the spatial configuration of fiducials {**X**}*_J_*; (2) TRE(**r**) can be expressed as the sum of two components: one related to uncertainty in position ***τ*** and the second related to uncertainty in the orientation data **R**; (3) both components of TRE(**r**) depend on the magnitude of the noise (TRE(**r**) increases for noisier fiducial measurements); (4) the orientation component is anisotropic; that is, it depends on the direction in space while the positional component is isotropic.

For the class of pose measuring systems which use point-based registration to track the pose of a rigid object, propagation of orientation uncertainty to a given POI is equivalent to the propagation of the fiducials’ uncertainty to a target point and, therefore, should inherit the above-mentioned characteristics of TRE(**r**). The anisotropic distribution of the orientation uncertainty was reported for the pose measuring system using a stereo camera to track spherical, reflective markers attached to an object [23]. It was found that the distribution is bimodal on a unit sphere with the smallest uncertainty located at poles defined by the eigenvector **e**_3_ associated with the largest eigenvalue of the covariance matrix of the orientation data. It was hypothesized that such a distribution offers an opportunity for better planning of robotic operations by ensuring that a given POI is in the region of small uncertainty. However, to take advantage of such a strategy, the direction of eigenvector **e**_3_ must stay fixed in the CAD coordinate frame regardless of the object’s orientation.

In this paper, we expanded the study in [23] to determine the conditions for which the observed behavior (stability of **e**_3_) holds by acquiring static measurements of several poses using a different pose measuring system (a large-scale tracking system iGPS). For each pose, the covariance matrix of the orientation data was determined. While the matrices were different for different poses, we found that the direction of the eigenvector **e**_3_ exhibited very small variations compared to the directions of the other two eigenvectors, **e**_1_ and **e**_2_, which showed larger variations. This behavior was reproduced in computer simulations and, to the best of our knowledge, it has not been reported in the literature. Analysis of existing theoretical expressions for TRE(**r**) in point-based registration reveals the reason for such unusual behavior of pose measuring systems which employ point-based registration to calculate 6DOF data. We found that misalignment between the directions of the anisotropic noise of fiducials and the directions of the axes of the moment of inertia characterizing the configuration of the fiducials is responsible for the observed phenomenon. It appears that the direction of **e**_3_ is almost independent of the misalignment whereas the directions of **e**_1_ and **e**_2_ were dependent on the misalignment. Furthermore, our study shows that **e**_3_ is well aligned with the eigenvector **b**_min_ of the moment of inertia matrix corresponding to the smallest eigenvalue.

The location of any POI is fixed relative to the locations of fiducials. Therefore, for the class of pose measuring systems discussed in this paper, if a vector pointing to a POI is aligned with **b**_min_, this POI will be in the region of small propagated uncertainty, regardless of the object’s orientation. Prior knowledge of such behavior may be useful in robotic operations when tight tolerances are required. A procedure for determining the placement of fiducials so that the smallest uncertainty is propagated to a given POI is introduced. The optimal placement of fiducials has been studied earlier for rigid body registration. Two main applied approaches were (1) use of theoretical models of TRE(**r**) [24] (some of them based on isotropic noise [25, 26]); (2) numerical search for the optimal placement using covariance matrices of experimental noise, evaluating transformations {**R**, ***τ***} and then corresponding to TRE(**r**) [22, 27]. While these studies showed implicit directional dependence of TRE(**r**) and its reduction, they did not alert practitioners that the uncertainty of a given POI on the rotated rigid object may depend on the object’s orientation nor provide clear guidance on how to ensure that this uncertainty will be close to the smallest possible value, regardless of object’s orientation. This paper attempts to provide this missing information.

In the next section, some background information and relevant equations are reviewed, followed by a brief description of the experimental setup and data postprocessing. This is followed by a presentation of the results, discussion, and conclusions.

## 2. Previous Research

In this section a brief review of the theoretical work relevant to our experiments is presented. Section 2.1 presents a brief review of point-based rigid body registration. This is followed by a discussion of the propagation of noise from the fiducials used to register two sets of points {**X**}*_J_* and {**Y**}**_J_** to the registration parameters {**R**, ***τ***} and then to the transformed target point; an analytical formula for TRE(**r**) based on anisotropic, homogenous Gaussian noise perturbing the fiducials is provided. In Section 2.2, the propagation of orientation uncertainty of a 6DOF rigid object to an individual point on its surface is discussed.

### 2.1. TRE in Point-Based Rigid Body Registration

Given two sets of *J* fiducials {**X**}*_J_* and {**Y**}**_J_** measured in the working and destination frames, respectively, the rotation **R** and translation ***τ*** which minimize the error function in (1) can be obtained in the following way. First, the origins of both frames are moved to the respective centroids **X**_avg_ and **Y**_avg_, that is, the locations of fiducials in the translated frames are **X̃***_n_* = **X***_n_* − **X**_avg_ and **Ỹ***_n_* = **Y***_n_* − **Y**_avg_, *n* = 1, …, *J*. Then, the covariance matrix **covXY_3×3_** is determined as

(2) $$\mathrm{covXY}=\frac{1}{J}\;\left[{\tilde{X}}_{1},\ldots ,{\tilde{X}}_{J}\right]\;{\left[{\tilde{Y}}_{1},\ldots ,{\tilde{Y}}_{J}\right]}^{T},$$

where [···]*^T^* is the transposed matrix. The rotation matrix **R** can be calculated as in [28]

(3) $$R={\mathrm{VDU}}^{T},$$

where the matrices **U** and **V** are obtained from the Singular Value Decomposition (SVD) of the covariance matrix

(4) $$\mathrm{covXY}={\mathrm{USV}}^{T},\;D=\left[\begin{array}{ccc} 1 & 0 & 0\\ 0 & 1 & 0\\ 0 & 0 & \det\;\left(VU^{T}\right) \end{array}\right].$$

Once the rotation matrix **R** is determined, the related translation vector ***τ*** is calculated as

(5) $$\tau =Y_{\text{avg}}-{\mathrm{RX}}_{\text{avg}}.$$

This transformation {**R**, ***τ***} minimizes FRE in (1) in the least-squares sense, and this procedure is implemented in many commercial software packages.

However, noise in the measured fiducials {**X**}*_J_* and {**Y**}*_J_* affects the registration transformation, and it needs to be propagated to the target **T***_X_* transformed to the destination frame, namely, **RT***_X_* + ***τ***. Intuitively, it is obvious that the statistical properties of the target error TRE(**r**) will depend on the characteristics of the noise perturbing the fiducial locations as well as on the location of the target relative to the configuration of the fiducials. Based on the seminal papers by Sibson [29] and Fitzpatrick et al. [18], most theoretical studies and supporting computer simulations split the registration {**R**, ***τ***} to two transformations: a “big” deterministic one {**R**_0_, ***τ***_0_} and a small noisy one {**ΔR**, **Δ*τ***}, that is, the two frames are first initially aligned using the big transformation and the fine tuning is done by the small rotation and translation. Thus, any point **x** in the working frame is transformed to **y** in the destination frame as

(6) $$y=\Delta R\;\left(R_{0}x+\tau_{0}\right)+\Delta \tau .$$

The rationale behind such an approach was put forward by Sibson who observed that the distribution of TRE was completely determined by stochastic noise in the fiducials and not by the big transformation {**R**_0_, ***τ***_0_}. This observation is an extension of the well-known property that a variance of a 3D point perturbed by Gaussian noise is the same in all coordinate frames related by any translation ***τ***, that is, **x_i_** → **x_i_** − ***τ***. As stated in [18], *“Neither this reorientation nor the special positioning of the origin above is necessary to effect a solution* […]*, nor for any part of the derivation that follows. However, they do reduce the complexity considerably, and they can be easily undone at the end.”* The big rotation **R**_0_ can be found from SVD of the covariance matrices **covXX** and **covYY** of fiducials {**X**}*_J_* and {*Y*}*_J_* as

(7) $$R_{0}=U_{Y}U_{X}^{T}$$

and the big translation ***τ***_0_ can by calculated by substituting **R** = **R**_0_ in (5). Since both matrices **covXX** and **covYY** are symmetric and have positive diagonal elements, their SVD decomposition yields

(8) $$\mathrm{covXX}=U_{X}\Lambda^{2}U_{X}^{T}$$

and similarly for **covYY.** Matrix **Λ**^2^ is diagonal matrix

(9) $$\Lambda^{2}=\left[\begin{array}{ccc} \Lambda_{1}^{2} & 0 & 0\\ 0 & \Lambda_{2}^{2} & 0\\ 0 & 0 & \Lambda_{3}^{2} \end{array}\right].$$

Matrix **covXX** is closely related to the matrix of the moment of inertia **M** as

(10) $$M=\text{trace}\;(\mathrm{covXX})\;I_{3\times 3}-\mathrm{covXX},$$

where **I**_3_**_×_**_3_ is the identity matrix. Thus, **Λ^2^** defines the moments of inertia relative to the three major axes, and the orientation of the axes is determined by matrix **U***_X_* in the working frame and **U***_Y_* in the destination frame. When a coordinate system is aligned with the axes of the moment of inertia (customarily done in theoretical analysis of TRE(**r**) in point-based rigid body registration) then matrix **M** takes a simple diagonal form

(11) $$M=\left[\begin{array}{ccc} \Lambda_{2}^{2}+\Lambda_{3}^{2} & 0 & 0\\ 0 & \Lambda_{1}^{2}+\Lambda_{3}^{2} & 0\\ 0 & 0 & \Lambda_{1}^{2}+\Lambda_{2}^{2} \end{array}\right].$$

It should be stressed that the moment of inertia characterizes the configuration of the fiducials in space, not the noise affecting the locations of the fiducials. In general, when the distance between fiducials is a few orders of magnitude larger than the noise, the moment of inertia relative to the major axes remains constant, that is, $\Lambda_{X}^{2}=\Lambda_{Y}^{2}$, and for this reason, we drop the subscript in **Λ**^2^.

While noise does not affect the moment of inertia, it has a great impact on the Target Registration Error (TRE(**r**)). Different forms for estimating TRE(**r**) were developed for different characteristics of fiducial noise, starting from the simplest isotropic, homogenous, Gaussian noise (the same for all fiducials) to the most complex, anisotropic, nonhomogenous Gaussian with nonzero mean (i.e., nonzero bias). No closed form solution has yet been developed for the most complex case. An analytical expression was provided for Gaussian, zero mean, homogenous, and anisotropic noise characterized by covariance matrix **Ψ**; see equation (51) in [30]. For such noise model, TRE(**r**) was evaluated from the variance var(**r**)

(12a) $$\mathrm{var}\;(r)=\frac{\text{trace}\;(\Psi )}{J}+{\left\|r\right\|}^{2}\;\alpha^{2}\;(u),$$

where **u** is the unit vector pointing towards the target **r**, that is, **r** = ||**r**||**u** and

(12b) $$\alpha^{2}\;(u)=\sum_{i=1}^{3}\sum_{j\neq i}^{3}\frac{u_{j}^{2}\;\left(\Lambda_{j}^{2}\Psi_{i,i}+\Lambda_{i}^{2}\Psi_{j,j}\right)}{{\left(\Lambda_{i}^{2}+\Lambda_{j}^{2}\right)}^{2}}+\sum_{i=1}^{3}\sum_{j\neq i}^{3}\sum_{k\neq i,k\neq j}^{3}u_{j}u_{k}\Psi_{j,k}\frac{\Lambda_{i}^{2}}{\left(\Lambda_{i}^{2}+\Lambda_{j}^{2}\right)\;\left(\Lambda_{k}^{2}+\Lambda_{i}^{2}\right)}$$

is the variance of the angular error (deviation of the directional vector **u**(*ϑ*, *φ*) from its nominal, noise free direction) and **u**(*ϑ*, *φ*) is parametrized by two spherical angles *ϑ* and *φ* as

(13) $$u\;\left(\vartheta ,\varphi \right)=[u_{1},u_{2},u_{3}]\;=[\cos\vartheta \cos\varphi ,\cos\vartheta \sin\varphi ,\sin\vartheta ].$$

Equation (12a) contains two terms: the first is isotropic and is related to the uncertainty in translation ***τ*** in (5); the second term is anisotropic as it depends on angles (*ϑ*, *φ*) and is related to uncertainty in the rotation **R** in (3). The isotropic term is inversely proportional to the number of fiducials *J*, and for most target locations which are not very close to the origin of the coordinate frame, the term related to orientation uncertainty in **R** will be dominant.

We note that the orientation of the noise matrix **Ψ** (i.e., the coordinate frame formed by its eigenvectors) and the orientation of the moment of inertia matrix **M** are completely unrelated and their relative orientation depends on the experimental conditions.

### 2.2. Propagation of Orientation Uncertainty of Rigid Body to a POI

Let vector **U** define the location of a POI in the CAD coordinate frame and let **u** be a unit vector parallel to **U** such that **U** = *U***u**. If **R***_j_* is the orientation matrix of a rigid object and **t***_j_* its location obtained from the *j*th measurement, then **U***_j_* is the location of the POI on the rotated object in the coordinate frame of the pose measuring instrument,

(14) $$U_{j}=Uw_{j}+t_{j},$$

where **w***_j_* is a unit vector pointing to a rotated POI in the coordinate frame of the instrument

(15) $$w_{j}=R_{j}u$$

and it can be parametrized by two spherical angles **w***_j_*(*ϑ_j_*, *φ_j_*) as in (13). We are interested in propagating the uncertainty of **R***_j_* to the uncertainty of **w***_j_*. We assume that

(16) $$R_{j}=R_{\text{avg}}\Delta R_{j},$$

where **R**_avg_ is the averaged orientation obtained from *N* repeated measurements acquired in the same experimental conditions, **ΔR***_j_* is a small random rotation (noise), and *j* = 1, …, *N*. In axis-angle representation (**a***_j_*, *ρ_j_*), the smallness of the rotation is gauged by small values of angle *ρ_j_* and this leads to the following expression for **ΔR***_j_* in linear approximation

(17) $$\Delta R_{j}\;\left(a_{j},\rho_{j}\right)\approx I+\left[\begin{array}{ccc} 0 & -q_{j}^{z} & q_{j}^{y}\\ q_{j}^{z} & 0 & -q_{j}^{x}\\ -q_{j}^{y} & q_{j}^{x} & 0 \end{array}\right],$$

where **I**_3_**_×_**_3_ is the identity matrix, **a***_j_* is a unit vector defining the axis of rotation, and

(18) $$q_{j}=\rho_{j}a_{j}.$$

A covariance matrix **C**(**q**) of the orientation data can be calculated as

(19) $$C\;(q)=\frac{1}{N}\;[q_{1},\ldots ,q_{N}]\;{\left[q_{1},\ldots ,q_{N}\right]}^{T}.$$

Repeated measurements of the orientation matrix **R***_j_* in (15) yield a corresponding set of vectors {**w***_j_*} which are tightly distributed around the average direction **w**_avg_. If *μ* denotes the angle between **w***_j_*(*ϑ_j_*, *φ_j_*) and **w**_avg_, then its distribution can be described by the Fisher-Bingham-Kent (FBK) distribution [31–33] as

(20) $$G_{\sigma ,\beta }\;(\mu )=\mu \sigma^{-2}\exp\;\left(\frac{-\mu^{2}}{2\sigma^{2}}\right)\;K_{\sigma ,\beta }\left(\mu \right),$$

where *σ* is the angular uncertainty and *K_σ_*_,_*_β_* is the Kent correction to the Fisher distribution

(21) $$K_{\sigma ,\beta }\;\left(\mu \right)=\frac{1}{2\pi }\sigma^{-2}\sqrt{\left(1-2\beta \sigma^{2})\;(1+2\beta \sigma^{2}\right)}\times \int_{0}^{2\pi }\exp\;\left(\beta \mu^{2}\cos2\eta \right)\;d\eta .$$

This correction takes into account the nonzero eccentricity parameter *β* which describes the shape of the elliptical contour of a constant probability on the (*ϑ*, *φ*) plane (*K_σ_*_,_*_β_* → 1 for symmetric circle contour when *β* → 0). Larger values of uncertainty *σ* correspond to larger deviations of vector **w***_j_* from the mean direction **w**_avg_. For pose measuring systems which use point-based rigid body registration, the angular uncertainty *σ* is equivalent to the angular uncertainty *α* from (12a) and (12b) when homogenous, anisotropic model of Gaussian noise characterizes the experimental conditions. However, the analysis in this subsection and as discussed here, the angular uncertainty *σ* is more general than the uncertainty *α* discussed in Section 2.1 because it is applicable to any sequence of noisy rotations **ΔR***_j_*, no matter what sensors and raw measurements were used to get the rotation matrices. Equations (12a) and (12b) are applicable only to the class of pose measuring systems which utilize point-based rigid body registration.

## 3. Data Collection and Processing

### 3.1. Experimental Setup

A commercially available, large-scale tracking system (iGPS) was used to collect 6DOF data [34]. The manufacturer specified positional uncertainty is 250 *μ*m. The system consists of a network of eight transmitters placed outside of the working volume (3m × 3m × 1.8 m) to track vector bars within the work volume. The transmitters were mounted on 3.05m high steel columns anchored to the concrete floor. The columns were evenly distributed around the perimeter of the lab space [15m × 16m × 10m (high)], and the working volume was in the center of the lab. Two vector bars were used in the experiment, and each vector bar contains two detectors which define a vector in space (the detectors in a vector bar were separated by 101.6mm). The two vector bars rigidly mounted to an aluminum rail were used to create a local coordinate frame: in commercial applications, a rigid object remains fixed in the local frame which is tracked by the system.

**Figure 1:**
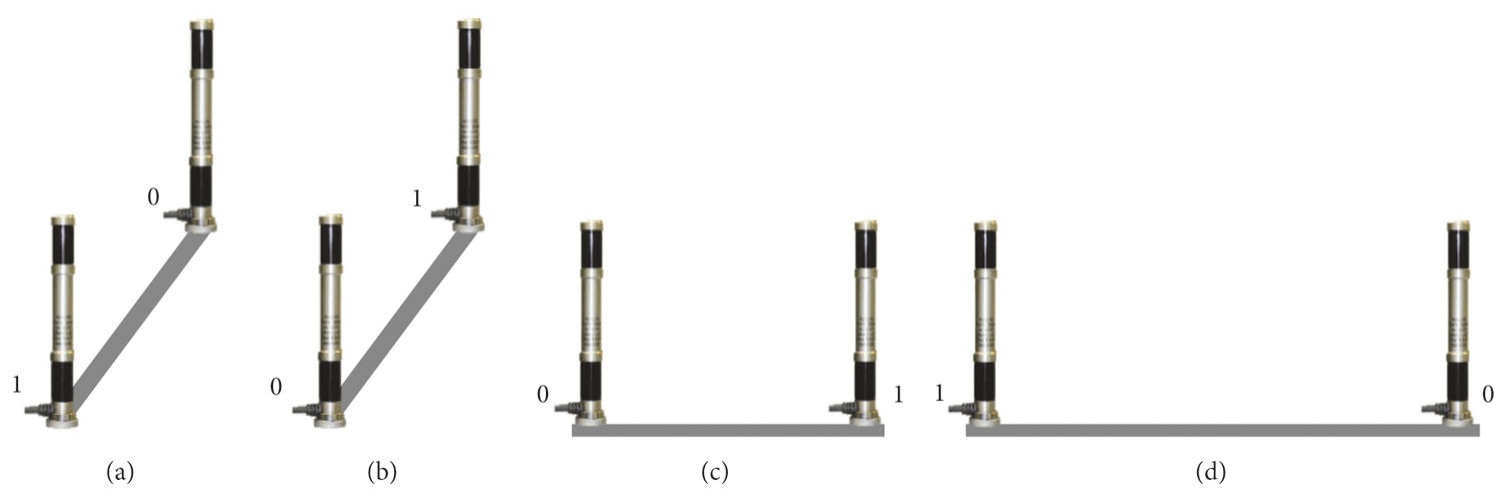
Four configurations (a, b, c, d) of the vector bars used in the experiments to create four different local frames.

Four different local frames were created and used to obtain measurements for four configurations of the vector bars; see Figure 1. Both vector bars were parallel to each other, and the distance between them was 375.7mm for configurations (a–c) and 902.2mmfor configuration (d). The line connecting the two bars for configuration (a) is parallel to that for (b) and similarly for configurations (c) and (d); the line in (a) and (b) is perpendicular to the line in (c) and (d). Each local frame was used to measure *M* different static poses (*M* = 12, 27, 12, 20 for frames (a–d), respectively), and at each pose, *N* repeated measurements in the same experimental conditions were acquired (*N* ≥ 50,000).

The system outputs 6DOF data (∠*X*, ∠*Y*, ∠*Z*, *x*, *y*, *z*) where the first three components are the angles of rotation. From the three angles, a rotation matrix **R** is constructed as

(22) $$R\;(∠X,∠Y,∠Z)=R_{X}(∠X)\;R_{Y}\;(-∠Y)\;R_{Z}\;(∠Z),$$

where **R***_X_*_,_*_Y_*_,_*_Z_* are matrices of the basic rotations around the axes of a fixed coordinate frame of the tracking system. The last three components of the 6DOF data are coordinates of the origin of the local frame defined by the user (a lower detector in the vector bar labeled as 0 in Figure 1). In addition to the 6DOF pose of the frame, the Cartesian coordinates of the four detectors constituting the two vector bars are also available. They were used as the locations of four fiducials for point-based rigid body registration in some of the computer simulations.

### 3.2. Data Postprocessing

For each *m*th pose, the averaged orientation **R**_avg_(*m*) was calculated from the repeated measurements or computer generated **R***_j_*, *j* = 1, …, *N*. There are different ways of calculating the average rotation matrix, and in this study, we used the mean rotation in the Euclidean sense. Specifically, **R**_avg_ was found as the orthogonal projection of a matrix $\bar{R}=1/N\sum_{j=1}^{N}R_{j}$; see equation (3.7) in [35]. Such a matrix retains the property of a rotation matrix and the expected properties of means of numbers, namely, invariance under permutation, biinvariance, and invariance under transposition. It also minimizes the error function based on the Frobenius norm; see [35] for details. Once **R**_avg_ was determined, the matrix of small random rotation was determined as

(23) $$\Delta R_{j}\;\left(a_{j},\rho_{j}\right)=R_{\text{avg}}^{T}R_{j}$$

from which the axis **a***_j_* and the angle *ρ_j_* were extracted. Axis angle representation of any rotation matrix has the following symmetry:

(24) R(a,-ρ)=R(-a,ρ).

In our calculations, we restricted the angle *ρ_j_* to always be positive and allowed the axis **a***_j_* to flip its direction to maintain the right-handedness of the coordinate frame. Once (**a***_j_*, *ρ_j_*) were known, the covariance matrix of the orientation data **C***_m_*(**q**) was calculated using (18) and (19) and its eigenvalues {Λ_1,_*_m_*, Λ_2,_*_m_*, Λ_3,_*_m_*} (where Λ_1_ < Λ_2_ < Λ_3_) and corresponding eigenvectors {**e**_1,_*_m_*, **e**_2,_*_m_*, **e**_3,_*_m_*} were evaluated for *m* = 1, …, *M*. Note that the inverse of large rotation $R_{\text{avg}}^{T}$ was already applied in (23) and, therefore, eigenvectors {**e**_1,_*_m_*, **e**_2,_*_m_*, **e**_3,_*_m_*} of the covariance matrix **C***_m_*(**q**) are determined in the CAD coordinate frame. In addition, the covariance matrix of positional data **C***_m_*(**Y**) and its eigenvectors and eigenvalues were determined where **Y** is the positional part of *N* repeated pose measurements in the instrument frame.

Histograms of the angular deviations *μ_j_* of the instantaneous unit vector **w***_j_*(*ϑ_j_*, *φ_j_*) from the corresponding unit mean vector **w**_avg_ were created for repeated registrations **R***_j_* and selected vectors **u** as follows from (15). Additionally, 2D histograms of the spherical angles (*ϑ_j_*, *φ_j_*) parametrizing the unit vectors **w***_j_* were constructed. For each set of unit vectors {**w***_j_*}, the corresponding parameters *σ* and *β* for the FBK distribution were determined as in [32]. These parameters were then used to generate the plot of the theoretical distribution *G_σ_*_,_*_β_*(*μ*) from (20). This theoretical FBK distribution was then compared with the histogram of the angles of deviation *μ_j_* obtained from the experimental data.

The evaluation of the angular uncertainty *σ* was repeated many times to get the distribution of *σ* on a unit sphere. For each average *m*th orientation, a grid of elevation and azimuth angles (*ϑ_i_*, *φ_l_*) was created, *i* = 1, …, *I*, *l* = 1, …, *L*, where *I* = 180 and *L* = 360, corresponding to angular increments of 1°. For each pair of angles, a unit vector **u***_i_*_,_*_l_*(*ϑ_i_*, *φ_l_*) parametrized as in (13) was defined, and all **R***_j_* rotations acquired for the *m*th pose were applied to **u***_i_*_,_*_l_* using (15). From the resulting set of unit vectors **w***_j_*(*ϑ_i_*, *φ_l_*), the corresponding angular uncertainty *σ*(*ϑ_i_*, *φ_l_*) was calculated as in [32]. The procedure was repeated for each vector **u***_i_*_,_*_l_*(*ϑ_i_*, *φ_l_*) in the *I* × *L* grid.

To show a link between the propagation of an object’s pose uncertainty to a POI and the propagation of uncertainty from fiducials to the target in registration problem, the distribution of the angular uncertainty *α*(*ϑ_i_*, *φ_l_*) predicted by (12a) and (12b) was determined using the same *I* × *L* grid of vectors **u***_i_*_,_*_l_*(*ϑ_i_*, *φ_l_*). For each dataset corresponding to **R**_avg_(*m*), the average locations of the four fiducials {**Y**_4_} were calculated. Then, the moment of inertia matrix **M** in (10) was evaluated and from its SVD decomposition, **Λ**^2^ in (9) was obtained and used in (12a) and (12b). In addition to **Λ**^2^, the noise covariance **Ψ** is required in (12a) and (12b). Since equation (12a) and (12b) can handle only homogenous noise, we arbitrary selected the covariance matrix **Ψ** of the first fiducial **Y**_1_. Once **Ψ** and **Λ**^2^ were determined, the angular uncertainty *α*(*ϑ_i_*, *φ_l_*) was calculated for each vector **u**(*ϑ_i_*, *φ_l_*) in the *I* × *L* grid.

As mentioned earlier, the orientation of matrix **M** is unrelated to the orientation of matrix **Ψ**. In (12a) and (12b), this is reflected by the fact that the coordinate frame can be rotated so that **M** is diagonal (only **Λ**^2^ is used in the equation) while noise **Ψ** is not (see off-diagonal elements Ψ*_j_*_,_*_k_* in the triple summation in (12a) and (12b)). To investigate the effect of relative misalignment between the two matrices, we performed SVD decomposition of the noise matrix

(25) $$\Psi =U_{\psi }\Psi_{0}U_{\psi }^{T}$$

and then replaced the original matrix with the rotated one

(26) $$\Psi \;(\omega )=\Omega \Psi_{0}\Omega^{T},$$

where rotation matrix **Ω**(**a**, *ω*) is determined by arbitrary axis **a** and angle of rotation *ω*. For *ω* = 0, **Ω** = **I**, **Ψ** = **Ψ**_0_, and both matrices **M** and **Ψ** are perfectly aligned. Larger angle *ω* corresponds to larger misalignment between **M** and **Ψ**. For each *ω*, the corresponding **Ψ**(*ω*) is used in (12a) and (12b) and the distribution of *α*(*ϑ_i_*, *φ_l_*) is recalculated.

## 4. Results

**Figure 2:**
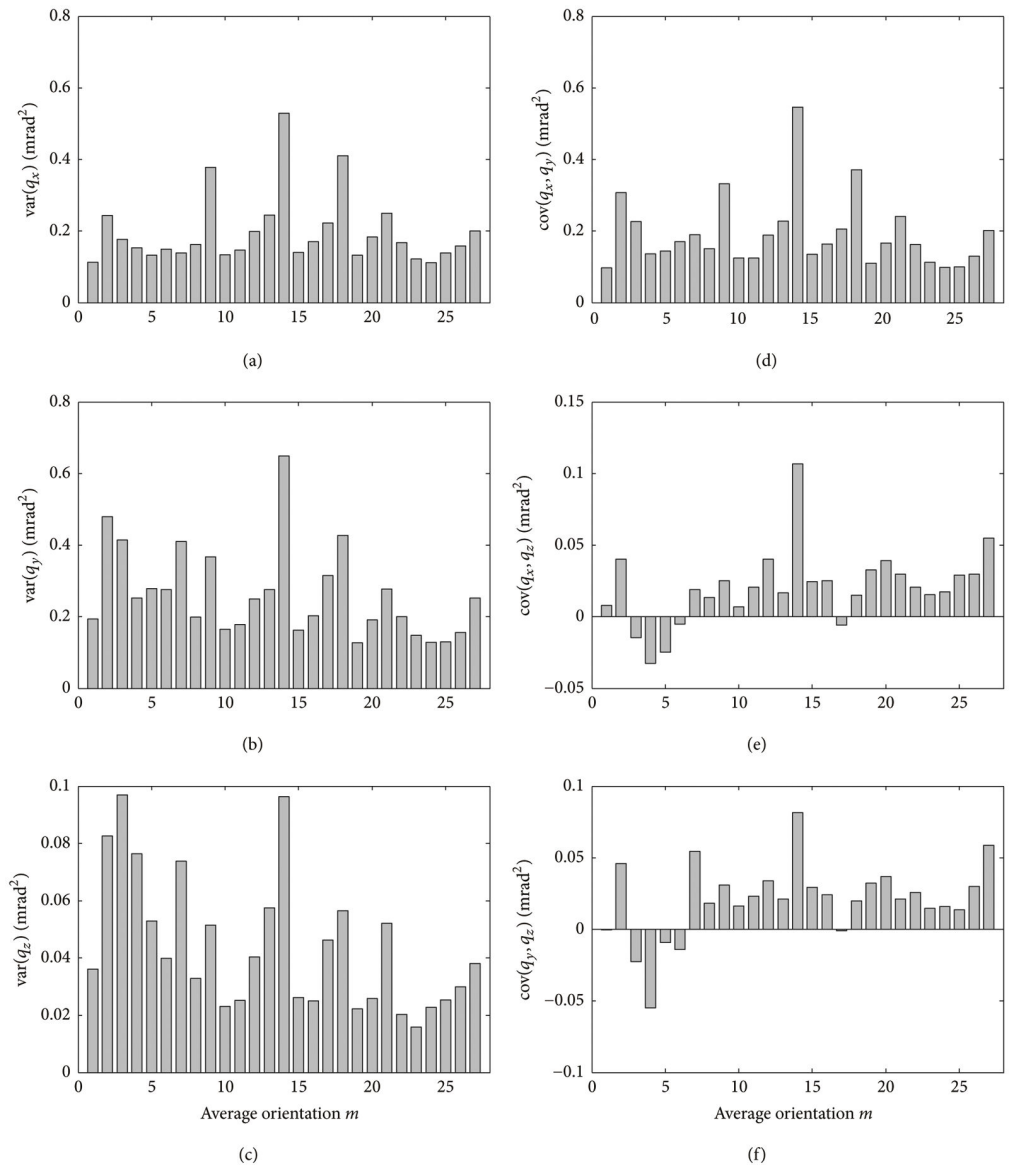
Elements of the covariance matrix **C***_m_*(**q**) for orientations **R**_avg_(*m*), *m* ≤ *M* = 27. Left column, plots (a–c): variances of the orientation data **q** (diagonal elements of covariance matrix); right column, plots (d–f): covariance coefficients of the orientation data **q**.

**Figure 3:**
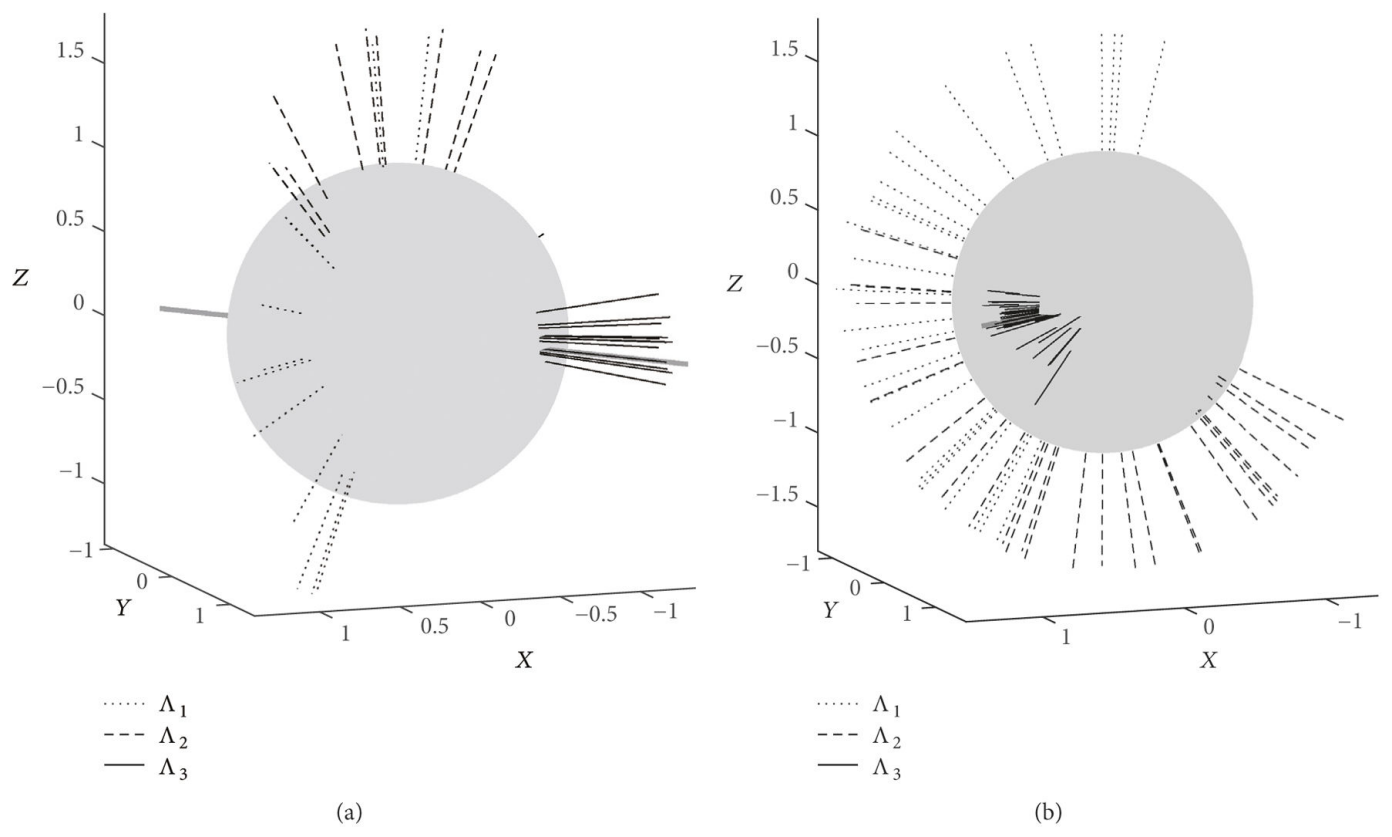
Spatial distribution of the three eigenvectors {**e**_1,_*_m_*, **e**_2,_*_m_*, **e**_3,_*_m_*} corresponding to the three eigenvalues {Λ_1,_*_m_*, Λ_2,_*_m_*, Λ_3,_*_m_*} of the covariance matrix **C***_m_*(**q**) for orientation **R**_avg_(*m*), *m* ≤ *M*: (a)*M* = 12, vector bars in configuration (a); (b)*M* = 27, vector bars in configuration (b). Both graphs are from the same view direction and the axes are unitless. Dotted lines denote eigenvectors **e**_1,_*_m_* associated with the smallest eigenvalue Λ_1,_*_m_* while solid lines are used to plot eigenvectors **e**_3,_*_m_* associated with the largest eigenvalue Λ_3,_*_m_*. The thick dark gray line indicates the direction of eigenvector **b**_min_ of moment of inertia matrix **M** for corresponding configuration of vector bars.

Figure 2 shows the elements of the covariance matrices **C***_m_*(**q**) calculated using (19) for each average orientation **R**_avg_(*m*), *m* ≤ *M* = 27 for vector bars in configuration (b). Figure 3 shows the spatial orientation of eigenvectors {**e**_1,_*_m_*, **e**_2,_*_m_*, **e**_3,_*_m_*} corresponding to the ordered eigenvalues {Λ_1,_*_m_*, Λ_2,_*_m_*, Λ_3,_*_m_*} of **C***_m_*(**q**) for configurations (a) and (b). Both graphs in Figure 3 are displayed from the same view angles. Eigenvectors corresponding to the largest eigenvalue Λ_3_ are shown as thick solid lines. Similar distributions of the eigenvectors were obtained for data acquired for vector bars in configurations (c) and (d) and in computer simulations with arbitrary configurations of vector bars.

The anisotropy of noise perturbing the locations of fiducials was checked by evaluating the ratio of eigenvalues Λ_3,_*_m_*/Λ_1,_*_m_* of the covariance matrices of positional data **C***_m_*(**Y**) for all datasets and the median value was equal to 3.27.

**Figure 4:**
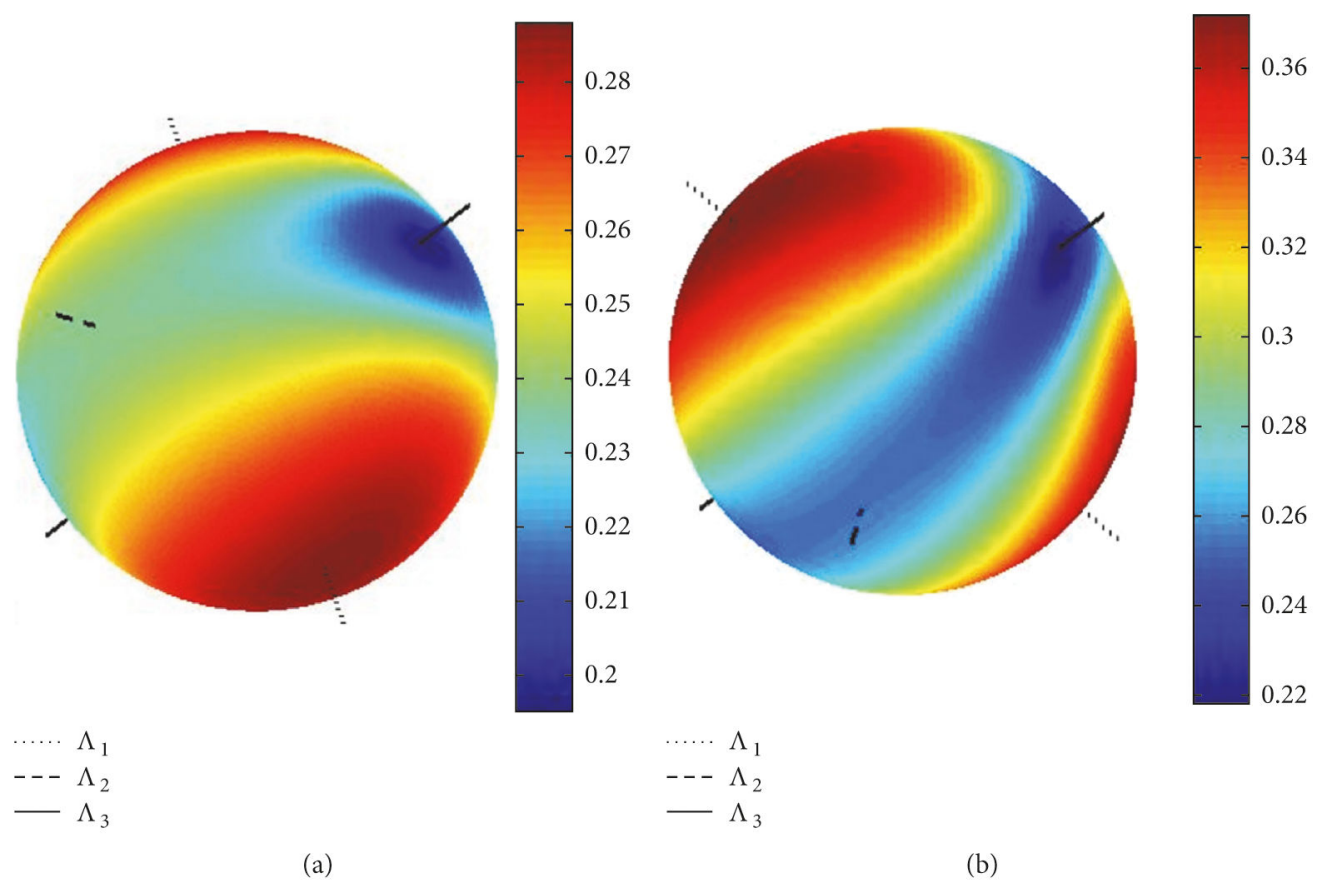
Directional distribution of the angular uncertainty *σ* in [mrad]. Both plots are shown from the same view angle. Directions of the three eigenvectors associated with eigenvalues {Λ_1_, Λ_2_, Λ_3_} of the covariance matrix **C***_m_*(**q**) (elements of which are displayed in Figure 2) are also plotted for: (a) *m* = 1; (b) *m* = 2.

**Figure 5:**
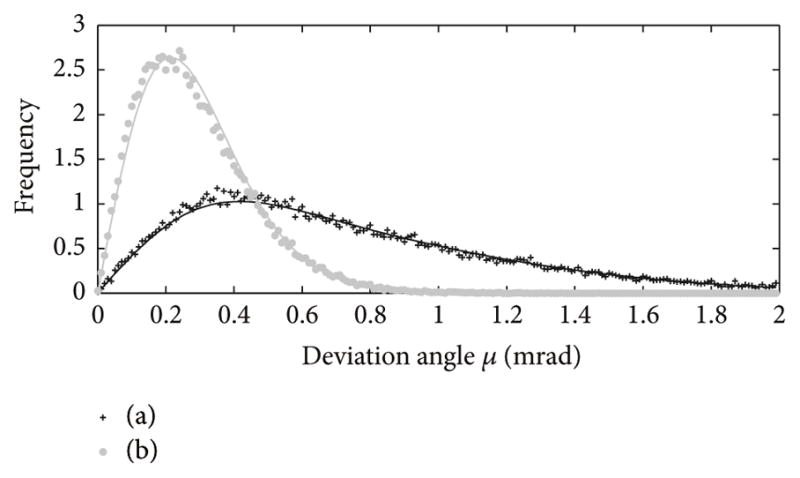
Histogram of angles *μ* (indicated by markers) and distribution *G_σ_*_,_*_β_*(*μ*) (lines) for two directions of **u**(*ϑ*, *φ*) where the angular uncertainty *σ*(*ϑ*, *φ*) shown in Figure 4(b) is equal to (a) *σ*_max_; (b) *σ*_min_.

The distributions of the angular uncertainty *σ* are shown in Figure 4 together with the directions of the corresponding eigenvectors {**e**_1,_*_m_*, **e**_2,_*_m_*, **e**_3,_*_m_*} of covariance matrix **C***_m_*(**q**) for *m* = 1 and *m* = 2(elements of **C***_m_*(**q**) are plotted in Figure 2). Histograms of the deviation angles *μ* and the associated FBK distributions *G_σ_*_,_*_β_*(*μ*) given by (20) are shown in Figure 5 for the same noisy orientation data used to create the plot in Figure 4(b). The angular uncertainty *σ* and eccentricity *β* were determined for the directions aligned with eigenvector **e**_1_ corresponding to *σ*_max_ and eigenvector **e**_3_ corresponding to *σ*_min_ in Figure 4(b). The values of *σ* were (0.37, 0.21) [mrad] and (2.86, 4.96) [mrad^−2^] for *β*.

**Figure 6:**
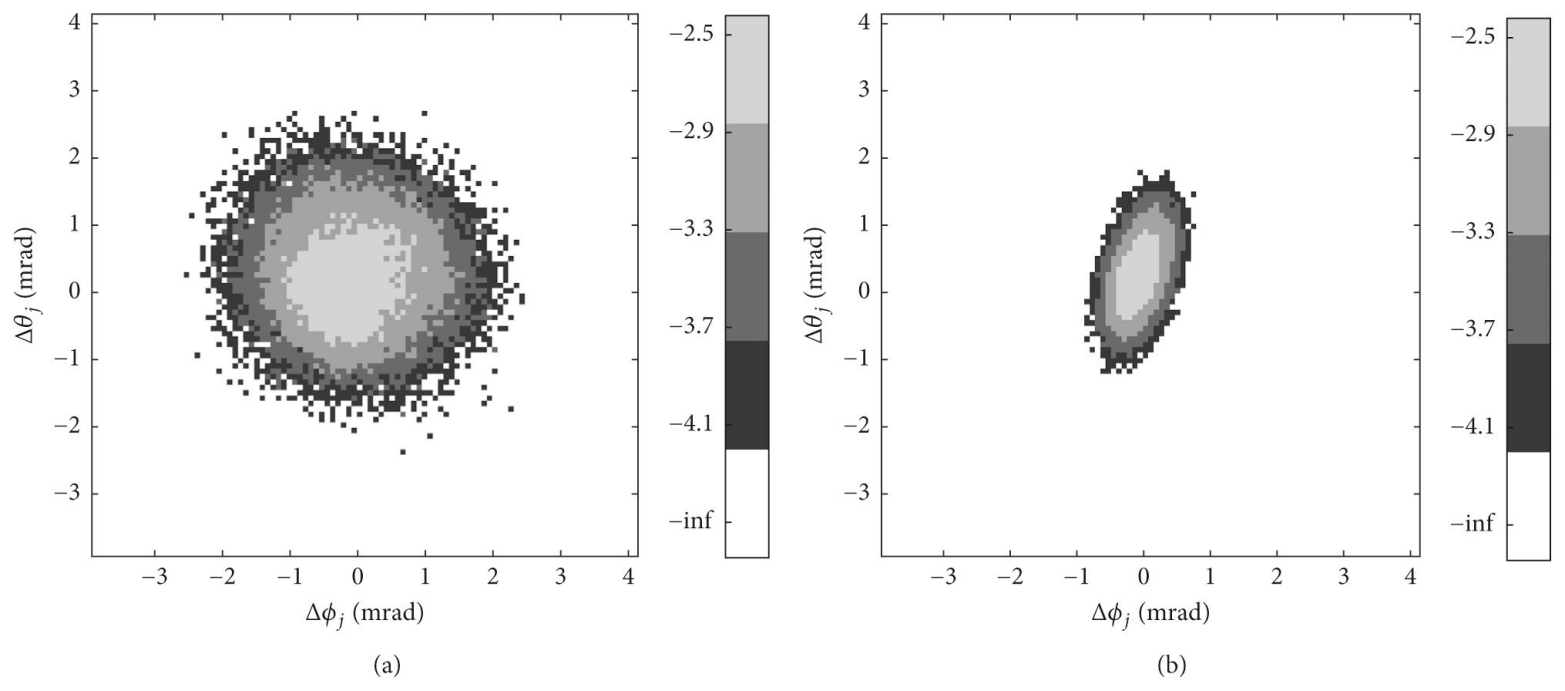
Histogram of angles (*ϑ_j_*, *φ_j_*) parametrizing unit vectors **w**(*ϑ_j_*, *φ_j_*) plotted in a log scale, where –inf indicates empty bins. The average unit vector **w**_avg_ (*ϑ*_avg_, *φ*_avg_) is aligned with the direction associated with (a) eigenvalue Λ_1_ from Figure 4(b) (related distribution *G_σ_*_,_*_β_* shown in Figure 5(a)); (b) eigenvalue Λ_3_ from Figure 4(b) (related distribution *G_σ_*_,_*_β_* shown in Figure 5(b)).

Figure 6 displays histograms of angles (*ϑ_j_*, *φ_j_*) parametrizing unit vectors **w***_j_*(*ϑ_j_*, *φ_j_*) in (15) for noisy rotations **R***_j_* used to create plots in Figures 4(b) and 5. Average vector **w**_avg_(*ϑ*_avg_, *φ*_avg_) is aligned with the direction where *σ*_max_ or *σ*_min_ are located in Figure 4(b), Δ*ϑ_j_* = *ϑ_j_* − *ϑ*_avg_ and Δ*φ_j_* = *φ_j_* − *φ*_avg_.

**Figure 7:**
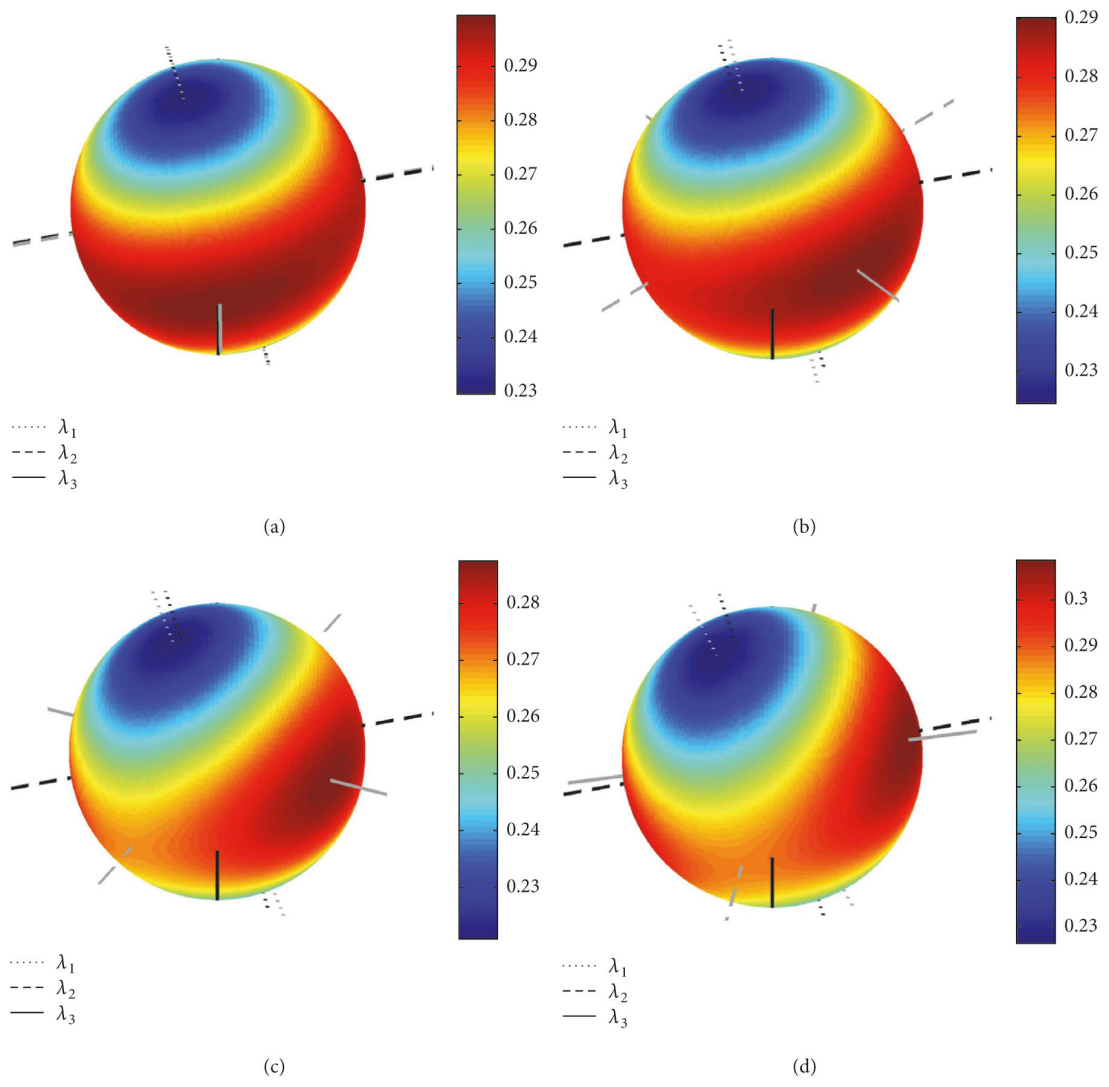
Distribution of uncertainty *α* in [mrad] for increasing misalignment angle *ω* in [rad]: (a) *ω* = 0.087; (b) *ω* = 0.785; (c) *ω* = 1.571; (d) *ω* = 2.356. Fixed eigenvectors for eigenvalues of matrix **M** (*λ*_1_ < *λ*_2_ < *λ*_3_) are plotted in black; the two major axes defined by locations of *α*_min_ and *α*_max_ and the third axis perpendicular to them are plotted in gray.

Finally, the plots in Figure 7 show examples of the distribution of angular uncertainty *α*(*ϑ*, *φ*) calculated using (12b) for increasing misalignment angle *ω* between the moment of inertia matrix **M** and the noise matrix **Ψ**. The plots were created for **Λ**^2^ = 10^4^ × diag([7.55, 1.02, 3.06]) [mm^2^], diagonal noise matrix (variances) in (26) **Ψ**_0_ = 10^−3^× diag([1.6, 2.2, 3.5]) [mm^2^], and the axis of rotation **Ω**(**a**, *ω*) in (26) is set arbitrary to **a** = [0.0384, 0.7319, 0.6804].

## 5. Discussion

The average orientations **R**_avg_(*m*) were acquired so that they differ substantially from each other. As expected, such wide variations in the poses result in large variations of the corresponding covariance matrices **C***_m_*(**q**). The three variances of the orientation data (diagonal elements of **C***_m_*(**q**)) and the three covariance coefficients (off-diagonal elements) shown in Figure 2 depend on the orientation **R**_avg_(*m*). The graphs in Figure 2 present data for vector bars in configuration (b), but similar variability in the elements of the covariance matrices **C***_m_*(**q**) was also observed for data acquired for configurations (a), (c), and (d). Despite this variability, the eigenvector **e**_3,_*_m_* which corresponds to the largest eigenvalue Λ_3,_*_m_* of the covariance matrix **C***_m_*(**q**) exhibits surprisingly weak dependence on the actual *m*th orientation for data acquired for a given configuration of vector bars; see solid lines in Figures 3(a) and 3(b). This is in striking contrast to the remaining two eigenvectors (marked with dashed and dotted lines). Note that eigenvectors **e**_3,_*_m_* are closely aligned with the direction of the eigenvector **b**_min_ corresponding to the smallest eigenvalue of the inertia matrix **M** given by (10).

The theoretical FBK distribution *G_σ_*_,_*_β_*(*μ*) as defined by (20) agrees very well with the experimental histogram of deviation angles *μ* as seen in Figure 5. Further evidence supporting this agreement can be seen in Figure 6. Kent postulated (see (1.6) in [32]) that the relation *βσ*^2^ → *c* (where 0 ≤ *c* < 1/2) holds for small noise. Indeed, the larger uncertainty *σ* and matching smaller eccentricity *β* in Figure 6(a) should be compared with the smaller *σ* and larger *β* in Figure 6(b). Recall that smaller eccentricity *β* describes a more rounded distribution. The difference between the distributions shown in Figures 6(a) and 6(b) will impact assembly tasks in manufacturing when tight tolerances are required.

The theoretical equations (12a) and (12b) derived for target registration error TRE(**r**) for point-based, rigid body registration are useful in explaining the propagation of pose uncertainty to a given POI (i.e., a target point). The target point remains fixed relative to the fiducials, that is, the target point relative to the major axes of the moment of inertia is fixed and independent of any imposed rotation of the object. However, when anisotropic noise affects the measurement of the fiducials, different “big” rotations disregarded in Sibson’s analysis of isotropic noise [29] will cause different orientations of the noise matrix relative to the fiducials. This will cause the angular uncertainty *α*(*ϑ*, *φ*) to be dependent on these rotations (note off-diagonal elements of noise matrix Ψ*_j_*_,_*_k_* in (12b) are affected by increasing misalignment angle *ω* in (26)), and the consequences of such dependence could be seen in Figure 7. The rotation of the gray axes in Figure 7 follows the pattern observed in the experiment; see Figure 3. Note in Figure 7 that the eigenvector **b**_min_ of matrix **M** corresponding to its smallest eigenvalue *λ*_1_ is well aligned with the direction defined by the two poles where *α* = *α*_min_, similarly to poles defined by *σ*_min_ and **e**_3_ in Figures 4 and 3. Exact matching between the theoretical *α* provided in (12a) and (12b) and experimental uncertainty *σ* is not expected since equation (12a) and (12b) was derived for anisotropic, homogenous noise (the same for all fiducials) while noise in experiment was anisotropic and nonhomogenous.

The surprising stability of eigenvector **e**_3_, pointing to *σ*_min_ in the CAD frame and the close alignment of **e**_3_ and **b**_min_, have a very useful implication. The direction of **b**_min_ depends solely on the selection of the fiducials, implying that the direction of **e**_3_ also depends on the selection of fiducials’ locations. Thus, if the location of a given POI associated with a rigid object is critical, it would be beneficial to place fiducials around an object in such a way that the axis of the smallest moment of inertia is parallel to the vector pointing towards this POI in the CAD frame. The principle for finding such configuration of fiducials is outlined in Appendix.

## 6. Conclusions

Many pose measuring systems derive pose from the measurement of fiducial markers attached to an object. The uncertainty of the fiducials’ locations propagates to the uncertainty of the object’s orientation which, in turn, propagates in an anisotropic way to individual points on the object’s surface. The angular distribution of the orientation uncertainty propagated to a POI depends generally on the object’s orientation. However, the orientation uncertainty in the regions close to the poles defined by the eigenvector corresponding to the smallest eigenvalue of the moment of inertia matrix of fiducials is almost independent of the object’s orientation. These regions are also characterized by the smallest propagated orientation uncertainty. Thus, strategic placement of fiducials around an object ensures that the orientation uncertainty propagated to a given POI is the smallest, regardless of the object’s orientation.

## Figures and Tables

**Figure 8:**
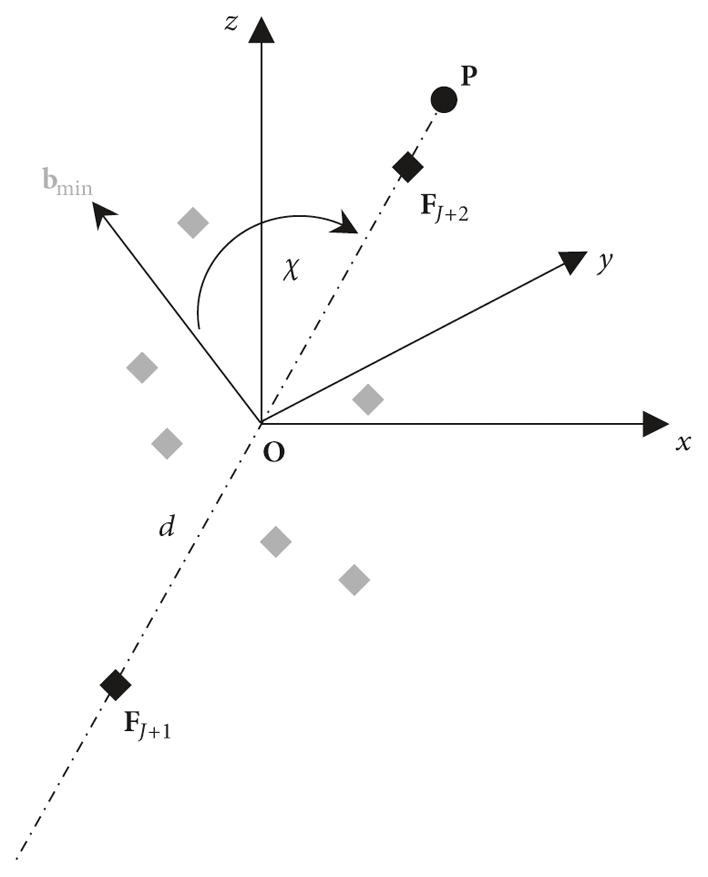
Optimal placement of fiducials for a given Point of Interest **P**. Gray diamonds indicate an initial configuration of *J* fiducials, **b**_min_ shows the direction of the principal axis corresponding to the smallest moment of inertia for the initial configuration, and two extra fiducials **F***_J_*_+1_ and **F***_J_*_+2_ are placed on line **OP** at a distance *d* on each side of center **O**. For sufficiently large *d*, the principal axis **b**_min_ of the new configuration for *J*+2 fiducials will be closely aligned with **OP** line.

**Figure 9:**
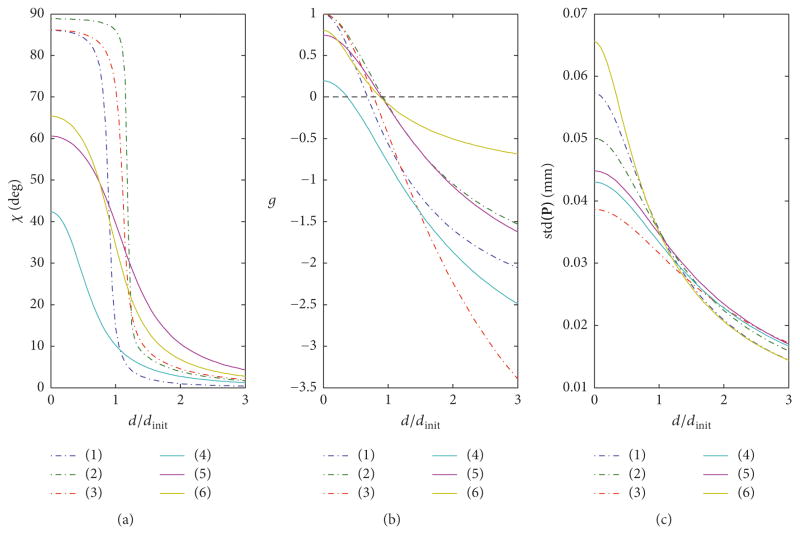
Results from computer simulations. Six random configurations of fiducials, lines (1–3) correspond to the worst-case selection of Point of Interest **P**: (a) angle *χ*; (b) normalized uncertainty *g* evaluated in (A.2); (c) standard deviation of **P** evaluated as square root of variance calculated in (12a).

## Appendix

The problem of finding optimal locations of fiducials for a given POI does not have a unique solution. In practical applications, the geometry of the rotated object and type of markers tracked by the instrument may impose additional constraints on possible fiducial locations. The general strategy for optimal placement is schematically shown in Figure 8 where gray diamonds depict an arbitrary configuration of *J* fiducials in the CAD frame.

In this general configuration, the principal axis of the smallest moment of inertia is denoted by **b**_min_, **P** is the location of a POI, and *χ* is the angle between **b**_min_ and **P**. We assume that the centroid **O** of *J* fiducials is located at the origin of the CAD frame; if not, the origin needs to be moved to coincide with **O** prior to determining line **OP**. To align **b**_min_ with line **OP**, two extra fiducials **F***_J_*_+1_ and **F***_J_*_+2_ are added and placed on line **OP** at a distance *d* on each side of center **O**. The added pair of fiducials does not change the location of centroid **O** and does not contribute to the moment of inertia about line **OP**. However, with increasing *d*, the two moments of inertia of the *J*+2 points about the two axes perpendicular to **OP** will increase. Thus, for a sufficiently large value of *d*, the moment of inertia about line **OP** will be the smallest and then **b**_min_, that is, the principal axis of the smallest moment of inertia evaluated for *J* + 2 points, will be closely aligned with line **OP**. This close alignment should cause the orientation uncertainty propagated to a given POI to be close to the minimum value.

To illustrate this procedure, numerical simulations were performed. Six different randomly selected configurations of *J* points (4 ≤ *J* ≤ 7) scattered within a cube of length 700mm were used. For each configuration, the covariance matrix **covXX** from (8) was determined and used to calculate the moment of inertia matrix **M** from (10) and its principal axes and the corresponding moments of inertia. Eigenvector corresponding to the smallest eigenvalue was determined as **b**_min_, and the initial distance *d*_init_, defined as the square root of the largest eigenvalue of **M,** was calculated using (11) for the initial configuration of *J* points

(A.1) $$d_{\text{init}}=\sqrt{\Lambda_{2}^{2}+\Lambda_{3}^{2}}.$$

Then, the covariance matrix of one of a positional data was chosen as a representative characteristic of noise affecting locations of fiducials and its version **Ψ** in the rotated frame aligned with eigenvectors of **M** matrix was evaluated. Then, the nondiagonal noise matrix **Ψ** and the three eigenvalues [ $\Lambda_{1}^{2},\Lambda_{2}^{2},\Lambda_{3}^{2}$] of **covXX** were substituted in (12b) to get the extreme values of the theoretical orientation uncertainty *α*, that is, *α*_max_(**u**_max_) and *α*_min_(**u**_min_). Finally, a Point of Interest **P** was selected. In the worst-case scenario, line **OP** was aligned with **u**_max_, that is, **P** had the largest uncertainty, and the misalignment angle *χ* between **b**_min_ and **OP** was close to 90°; in the general case, an arbitrary point **P** was selected, and the corresponding angle *χ* was less than 90°. Then, a pair of points **F***_J_*_+1_ and **F***_J_*_+2_ was added and the distance *d* varied from zero to 3*d*_init_. For each *d*, the moment of inertia matrix **M** was determined using all *J* + 2 points, and the corresponding eigenvector **b**_min_(*d*), angle *χ*(*d*), and the smallest theoretical uncertainty *α*_min_(*d*) were evaluated.

Figure 9 shows representative graphs from the six simulations. Parameter *g*(*d*), displayed in Figure 9(b), is the normalized uncertainty defined as

(A.2) $$g\;(d)=\frac{\left[\alpha_{\min}\;(d)-\alpha_{\min}\;(0)\right]}{\left[\alpha_{\max}\;(0)-\alpha_{\min}\;(0)\right]}.$$

For the worst-case selection of POI **P**, *g*(0) = 1, while for a generic location of **P**, 0 < *g*(0) < 1. Negative values of *g*(*d*) indicate that the smallest propagated uncertainty *α*_min_ for the optimal configuration of *J*+2 fiducials (which contains a pair of added fiducials separated by the distance 2*d*) is smaller than the smallest uncertainty *α*_min_ for the initial configuration of *J* fiducials. The uncertainty of the location of **P** (including both positional and rotational uncertainty propagated from the 6DOF pose) was calculated using (12a), and to suppress the trivial dependence of variance on length of **P**, a constant ||**P**|| = 350mm was used in all six simulations.

As seen in Figures 9(b) and 9(c), the required distance *d* should be close to *d*_init_. If the two extra fiducials cannot be placed at the distance *d*_init_ from **O** due to geometrical constrains, then the general procedure outlined above needs to be modified; for example, two pairs of fiducials (**F***_J_*_+1_, **F***_J_*_+2_ and **F***_J_*_+3_, **F***_J_*_+4_) could be placed on line **OP** at equal distances *d*_1,3_ < *d*_init_ and *d*_2,4_ < *d*_init_.
